# Androgen Receptor: Evaluation and Correlation with Recurrence and Clinicopathological Parameters in Papillary Urothelial Carcinomas of the Urinary Bladder

**DOI:** 10.7759/cureus.6715

**Published:** 2020-01-20

**Authors:** Shazia Mumtaz, Zubaida Hussain, Taimoor K Janjua, Atif Ali Hashmi, Saad Saleem Qureshi, Muhammad Usman Tariq, Naveen Faridi

**Affiliations:** 1 Pathology, Dow University of Health Sciences, Karachi, PAK; 2 Pathology, Liaquat National Hospital and Medical College, Karachi, PAK; 3 Pathology, Aga Khan University Hospital, Karachi, PAK

**Keywords:** androgen receptor, urothelial carcinoma, low grade papillary urothelial carcinoma, high grade papillary urothelial carcinoma, recurrence

## Abstract

Papillary urothelial carcinoma (PUC) is the most common malignant tumor of the urinary bladder. Urothelial tumors are notorious for frequent recurrences and follow a chronic relapsing course in most of the patients. In Pakistan, the incidence of PUC is showing a rising trend. Various immunohistochemical (IHC) markers including androgens have been studied as prognostic and predictive markers in PUC with conflicting results. Androgen is a steroid-based sex hormone and plays an important role in different body organs such as urinary bladder, prostate, muscles, and brain. We aimed to investigate the role of the IHC expression of androgen receptor (AR) as a predictor of recurrence in papillary urothelial carcinoma patients. Eighty-four patients were included in the study. Tissues from the biopsy specimens of these cases were stained with antibodies against AR; 17% of the cases demonstrated a positive AR IHC expression. The expression was slightly more common in low-grade carcinoma. The AR expression was not significantly associated with clinicopathological features. Recurrence was observed in 49% of the cases, and it was significantly more common in AR-negative cases (p-value: 0.025). Eighteen out of 19 patients who died of disease were AR- negative, but no statistical significance was observed. We conclude that the IHC expression of AR can be used as a predictive marker for PUC as it correlates with the recurrence rate.

## Introduction

Papillary urothelial carcinoma (PUC) is the most common malignant tumor of the urinary bladder, comprising 90% of all primary bladder carcinomas. It is more common in males than in females [1,2]. In the US, 80,470 new cases of bladder cancer and 17,670 deaths from bladder cancer were reported in 2019 [1]. Multiple markers have been investigated as diagnostic and prognostic indicators for urothelial carcinomas [3,4,5]. Androgen is a steroid-based sex hormone, and it plays an important role in different body organs such as the urinary bladder, prostate gland, muscles, and the brain [6-8]. Androgen is one of the markers whose expression can be seen in normal urothelial mucosa as well as carcinomas of the urinary bladder [9].

According to Globacon 2018, bladder cancer ranks eighth among all malignancies in Pakistan, with 4,610 new cases and 2,614 deaths reported in 2018 [10]. Bladder cancers carry high recurrence rates (10-year recurrence rate: 74.3%), and hence the patients need life-long monitoring [11]. Urothelial cancers top the list of tumors that cause the most economic burden since they reportedly incur the highest lifetime costs per patient among all malignancies [12]. As a result, further studies are urgently required to develop markers, which can aid in the separation of cases that are less likely to recur and thus contribute to the reduction of treatment costs. The role of the androgen receptor (AR) status as a predictive marker of PUC has been assessed in different populations worldwide [13-21]. However, no such assessment has been performed in the Pakistani population to our knowledge. This paucity of data relating to the Pakistani population prompted us to investigate the correlation of AR expression with recurrence in urothelial tumors in patients at a hospital in Karachi, Pakistan.

## Materials and methods

Study population and settings

This retrospective study was conducted on patients diagnosed with low- or high-grade PUC between July 2009 and June 2019. In all cases, follow-up data was determined through data available for at least one year for patient survival or reported deaths from carcinoma, depending on whichever came first. The study was conducted at the Department of Histopathology, Liaquat National Hospital and Medical College, Karachi, Pakistan. Institutional board approval was taken from the Research and Ethics Committee of the hospital.

Methodology

The surgical pathology slides at the Department of Pathology reported as PUC during the study period and paraffin blocks were reviewed. The hematoxylin and eosin section for each case was reviewed by two pathologists for confirmation of the original diagnosis based on the 2004 WHO/International Society of Urological Pathology Classification. In selected cases, immunohistochemistry (IHC) was performed using a monoclonal mouse anti-AR antibody on paraffin-embedded tissue sections of transurethrally resected tissue samples. The Dako REAL EnVision Detection System, Peroxidase/DAB, Rb/Mo (Dako, Denmark) was used for IHC staining. Briefly, 5-µm serial sections were cut for formalin-fixed, paraffin-embedded (FFPE) tissue onto Superfrost slides (Thermo Scientific, Germany). The sections were deparaffinized in xylene and rehydrated in graded series of ethanol (Merck, Germany). Heat-induced antigen retrieval was performed in a 10-mM citrate buffer (PH6.0) for one hour in a boiling water bath. Endogenous peroxidase activity was blocked by immersing slides in 0.3% w/v H2O2 room temperature for 10 minutes. Next, the antihuman AR antibody (mouse monoclonal IgG, clone AR 441; Dako diluted 1:50) was applied for four hours at room temperature. After three washes for five minutes each in phosphate-buffered saline (PH7.4), horseradish peroxidase (HRP)-labeled secondary antibody was applied for one hour at room temperature. After washing, the substrate was added and DAB was used for visualization. Hematoxylin was used for counterstaining. The primary antibody was replaced by phosphate-buffered solution and prostatic tissue served as negative and positive controls respectively.

Scoring

The AR expression was assessed by counting 500 tumor cells in the most immune-reactive area of the slides. The AR expression was considered positive when >10% of the tumor cells showed nuclear staining [13].

Statistical analysis

The Statistical Package for Social Sciences version 20 (SPSS; IBM, Armonk, NY) was used to analyze data. Frequencies and percentages were computed for categorical variables. Mean and standard deviations were computed for quantitative variables. A chi-square test was applied to investigate the association of categorical variables. The odds ratio (OR) was calculated by univariate binary logistic regression. A p-value of ≤0.05 was considered statistically significant. Overall survival was taken as the time from surgical excision till death or the last follow-up, and disease-free survival was defined as the time between surgical excision and local recurrence or distant metastasis, death, or the last follow-up.

## Results

A total of 84 PUC patients were included in our study, of which 66 were males (78.6%) and 18 females (21.4%). The mean age was 64.30 (±15.34) years (range: 8-92 years). Both low- and high-tumor grade PUC groups comprised 42 patients each. The mean follow-up duration was 13.32 (±10.55) months (range: 1-40 months). Recurrence was defined as the appearance of new growth in the bladder and/or ureter at least six months after the complete removal of initial growth. The recurrent growth was histologically confirmed to be PUC. Recurrence was noted in 41 (48.8%) patients. In our study, positive staining of AR was observed in 14 cases (16.7%). Detailed clinicopathological features of patients are presented below (Table 1).

**Table 1:** Clinicopathological profile of patients *Figures in parentheses represent standard deviation

	Mean
Age, years	64.30 (±15.34)*
Follow-up, months	13.32 (±10.55)*
Recurrence, months	12.78 (±10.35)*
Gender, n (%)	
Male	66 (78.6)
Female	18 (21.4)
Tumor grade, n (%)	
Low	42 (50)
High	42 (50)
Deep muscle invasion, n (%)	
Present	10 (11.9)
Absent	74 (88.1)
Androgen receptor expression, n (%)	
Positive	14 (16.7)
Negative	70 (83.3)
Recurrence, n (%)	
Yes	41 (48.8)
No	43 (52.2)
Survival status, n (%)	
Alive	65 (77.4)
Dead	19 (22.6)

Among AR-negative patients, recurrence was noted in 38 (58%) patients, while among AR-positive patients, recurrence was observed in only 3 (21.4%) patients. We found a significant association between AR-receptor negativity and recurrence (p: 0.025). AR was positive in 8 (19%) low-grade and 6 (14.3%) high-grade carcinomas; there was no significant association between AR expression and tumor grade (p: 0.558) (Table 2).

**Table 2:** Association between androgen receptor status and recurrence *Statistically significant

		Androgen receptor status, n (%)		P-Value
		Positive	Negative	
Recurrence	Yes	3 (21.4)	38 (54)	0.025*
	No	11 (78.6)	32 (45)	
Tumor grade	Low	8 (19)	34 (81)	0.558
	High	6 (14.3)	36 (85)	

No significant difference was found in AR-positive expression with respect to gender, lamina propria invasion, or deep muscle invasion (Table 3).

**Table 3:** Parameters showing independent predictors of androgen receptors

		Frequency, n (%)	Odds ratio	P-value	95% CI
Age groups					
	≤50 years	17 (20.2)	1		
	>50 years	67 (79.8)	0.26	0.210	0.032–2.139
Gender					
	Male	66 (78.6)	1		
	Female	18 (21.4)	1.778	0.480	0.360-8.784
Tumor grade					
	Low	42 (50)	1.412	0.559	0.444–4.493
	High	42 (50)	1		
Invasion In lamina propria					
	Present	70 (83.3)	1.292	0.764	0.244–6.849
	Absent	14 (16.7)	1		
Deep muscle invasion					
	Present	10 (15.5)	2.711	0.149	0.700–10.504
	Absent	74 (84.5)	1		
Recurrence					
	Yes	41 (48.80)	0.230	0.034	0.059–0.895
	No	43 (51.20)	1		

 Deaths occurred in 19 cases, of which 18 cases were AR-negative. Recurrence was less likely to develop in females in comparison to males (OR: 0.321), while older patients were more likely to develop recurrence in comparison with younger patients (OR: 2.78) (Table 4).

**Table 4:** Odds ratio showing independent predictors of recurrence CI: confidence interval

		Frequency, n (%)	Odds ratio	P-value	95% CI
Age groups					
	≤50 years	17 (20.2)	1		
	>50 years	67 (79.8)	2.787	0.080	0.884–8.789
Gender					
	Male	66 (78.6)	1		
	Female	18 (21.4)	0.321	0.050	0.103–1.002
Tumor grade					
	Low	42 (50)	1.100	0.827	0.467–2.589
	High	42 (50)	1		
Invasion In lamina propria					
	Present	10 (15.5)	0.947	0.936	0.253–3.549
	Absent	74 (84.5)	1		
Deep muscle invasion					
	Present	10 (15.5)	0.543	0.323	0.162–1.822
	Absent	74 (84.5)	1		
Androgen receptor					
	Present	14 (16.7)	0.230	0.034	0.059–0.895
	Absent	70 (83.3)	1		

## Discussion

AR is a ligand inducible transcription factor that mediates the biological effects of androgen hormone [14]. Androgen hormone is a sex-steroid hormone present in most body organs including the urinary bladder [9]. Multiple studies have investigated the association between androgens and bladder cancer pertaining to different aspects including tumor grade, recurrence rate, and clinical stage [4,5,15]. Papillary urothelial carcinomas are male-predominant carcinomas and this observation has also been highlighted in various studies, indicating the role of androgen in urothelial carcinomas [5,16].

In Pakistan, bladder cancer incidence is rising [10]. In our study, we evaluated the AR expression in bladder cancer patients and found that AR-positive expressions correlated with decreased risk of recurrence. We also found higher recurrence in males and the elderly.

Androgen hormone-binding activates AR leading to the progression of bladder cancers [17]. Androgen hormone downregulation by using anti-androgens has a protective effect in PUC. In a study done on patients diagnosed with prostate carcinoma, androgen-deprivation therapy was given by androgen receptor blockers, resulting in decreased androgen hormone levels. With long-term follow-ups, it was observed that androgen deprivation decreased the incidence of bladder cancer [18].

Androgen hormone and its receptor have been found to be strongly associated with bladder cancer progression and tumorigenesis [17,19,20]. In a large multinational cohort study, no association was found between the loss of AR and clinical outcomes. Investigators performed a study at two different city hospitals and, in both the centers, no association of AR was noted with respect to tumor grade, type, stage, and gender of patients [15,21]. The loss of AR expression was seen in high-grade urothelial cancers and invasive tumors, while no association was noted between AR expressions and tumor recurrence [13].

A multi-institutional based study on a large number of patients compared tumor grade with androgen status. It found no association between tumor grade and AR status [15]. This was similar to other findings in our study, while another study reported a negative association between AR status and tumor grade and invasiveness [4].

In our opinion, such varied results in studies can be attributed to multiple factors, such as sampling and laboratory techniques. In sampling techniques, factors that can influence could include sample size and genetic make-up of the population being studied. The laboratory factors that can contribute include the fixation time of tissues in formalin, the use of antibodies from different companies, methods of dilution, different cut-off parameters used for positive and negative results, and IHC vs molecular-based techniques.

Urinary bladder carcinomas belong to a group of tumors that have a high recurrence rate; therefore, regular surveillance with urine cytology and cystoscopy is an essential part of treatment protocol [22]. There is an urgent need to develop markers that can serve as prognostic and predictive indicators in bladder tumors. As regular surveillance is costly, the AR status can aid in this regard for patient selection. Going forward, the AR status can be used to give endocrine therapy in selected patients [23]. We recommend larger studies to establish correlations with staging and concurrent application of IHC along with other molecular-based techniques.

Our study strengths include a large sample size and patient follow-up. Limitations were the usage of IHC methodology and transurethral resection samples.

## Conclusions

We are able to conclude that positive expression of AR correlates with decreased recurrence rate in PUC, irrespective of histologic grade. AR can be utilized as a new predictive marker in urothelial carcinomas, especially in developing countries.
